# A bibliometric analysis of global trends in the research field of pharmaceutical care over the past 20 years

**DOI:** 10.3389/fpubh.2022.980866

**Published:** 2022-10-17

**Authors:** Yu Wang, Yifei Rao, Yuling Yin, Yaolei Li, Zhijian Lin, Bing Zhang

**Affiliations:** ^1^School of Chinese Materia Medica, Beijing University of Chinese Medicine, Beijing, China; ^2^Center for Pharmacovigilance and Rational Use of Chinese Medicine, Beijing University of Chinese Medicine, Beijing, China

**Keywords:** bibliometric analysis, pharmaceutical care, clinical pharmacy, developmental trends, research frontiers

## Abstract

Pharmaceutical care is essential in building up the basics of public health and clinical care. A comprehensive understanding of global status in the field of pharmaceutical care is necessary for directing its research frontiers and future trends. Therefore, this study aims to make a bibliometric analysis to track the development of pharmaceutical care research worldwide during the past two decades. The publications regarding pharmaceutical care were culled from the Web of Science Core Collection (WoSCC). Countries, institutions, authors, journals, references, and keywords in this field were visually analyzed by using VOSviewer (version 1.6.17) and CiteSpace (Version 5.8.R3). As a result, 3,597 publications (3,177 articles and 420 reviews) were obtained. The annual yields grew more than three times in the past two decades, from 54 records in 2002 to 379 papers in 2021. The United States played the leading role in this research from multiple aspects, including publication (*n* = 1,208), citations (*n* = 28,759), funding agencies, and collaboration worldwide. The University of Sydney in Australia was the most contributed institution with the greatest number of publications (*n* = 112) in pharmaceutical care research. Hersberger KE from the University of Basel was the most productive author (*n* = 40). Chen TF from the University of Sydney was the author who owed the highest H-index of 19 and most citations (*n* = 1,501). They both significantly impacted this field. American Journal of Health System Pharmacy produced the most publications, while Pharmacotherapy had the highest IF (IF_2020_ = 4.705) in this field. Clusters networks of co-cited references and keywords suggested that clinical pharmacy is an essential theme in pharmaceutical care. Terms of medication safety and critical care recognized by burst analysis of keywords also hint at the recent attention on clinical pharmacy. The present bibliometrics analysis may provide a comprehensive overview and valuable reference for future researchers and practitioners in the research field of pharmaceutical care.

## Introduction

Pharmaceutical care is defined as the responsible provision of pharmaco-therapy to achieve definite outcomes that improve or maintain a patient's quality of life” (1). It is a continuous quality improvement process for the use of medicinal products. The concept of pharmaceutical care has actually evolved over time. Its prototype term was from the “pharmacist care activities” in the 1960s (2). As to 1990s, the term of pharmaceutical care first emerged in the United States and then extended to other countries quickly (3–5). Meanwhile, a large number of other terms also appeared to describe “pharmacist care activities,” including “medication therapy management,” “pharmaceutical assistance,” and “pharmacy services,” Over recent decades, the considerable rise in mortality associated with the increasing complexity of medicine use has obtained great attention worldwide (6). For example, The World Health Organization (WHO) and the International Pharmaceutical Federation (FIP) were published the handbook on Developing pharmacy practice—A focus on patient care, which proposed that pharmaceutical care was aim at optimizing patient outcomes and benefiting the effective, rational and safe use of medicines (7). Thus, pharmacists have been faced with increasing health demands more than selling medicines, which forced the evolution of the pharmacist's role from product-oriented to patient-oriented services (8).

People are committed to developing advanced pharmaceutical care, progressively becoming vital within developed healthcare systems and contributing to positive health and economic outcomes (9, 10). As far as the content of basic pharmaceutical care, from traditional pharmaceutical care (involving dispensing, counseling, distribution, storage, and procurement) to enhanced pharmaceutical care (including prescription monitoring and drug utilization review, pharmaceutical care, pharmacovigilance, pharmaco-economics, services at drug information centers and poison control centers) (11). The responsibilities of pharmaceutical care are helping patients to make the best use of their medicines, and providing more services and care to help meet the demand for convenient, accessible, and cost-efficient health care services (12). The intervention outcomes include economics, health-related quality of life, patient satisfaction, medication appropriateness, adverse drug events, and adverse drug reactions (13). In recent years, pharmaceutical care has shown great potential and value, including hypertension, hyperlipidemia, diabetes, asthma, and chronic obstructive pulmonary disease, medication review and management, and prescribing assessment (14–16). It confirms the convergence between high-interest priority areas identified by government stakeholders and the health and economic evidence of pharmaceutical care. This convergence will improve both health impacts and overall system sustainability. Consequently, it is significant to analyze the current status quo, focus areas, and future prospects related to pharmaceutical care.

Bibliometric analysis is a valuable method to quickly reveal the developmental status and research frontiers in a specific field (17). It has recently been considered an interdisciplinary science based on statistical and visualization techniques and has been widely used in many scenarios. For instance, a bibliometric analysis of research on multiple criteria decision-making depicted its developmental status and revealed its research focus in different periods (18). Another 30-year bibliometric study demonstrated the research trends of fuzzy theory in China (19). And bibliometric analysis also has been applied in the medical field (20). Through the literature search, we noticed a rapid increase in publications concerning pharmaceutical care in nearly 20 years, which indicates a growing awareness from scholars in this field. However, a bibliometric analysis of pharmaceutical care during this period is yet to be seen. Therefore, the present study performed a bibliometric analysis to understand the current status and frontiers of pharmaceutical care. We hope it will provide definite directions and valuable references for further research in pharmaceutical care.

## Materials and methods

### Search strategies and data collection

**Figure 1:**
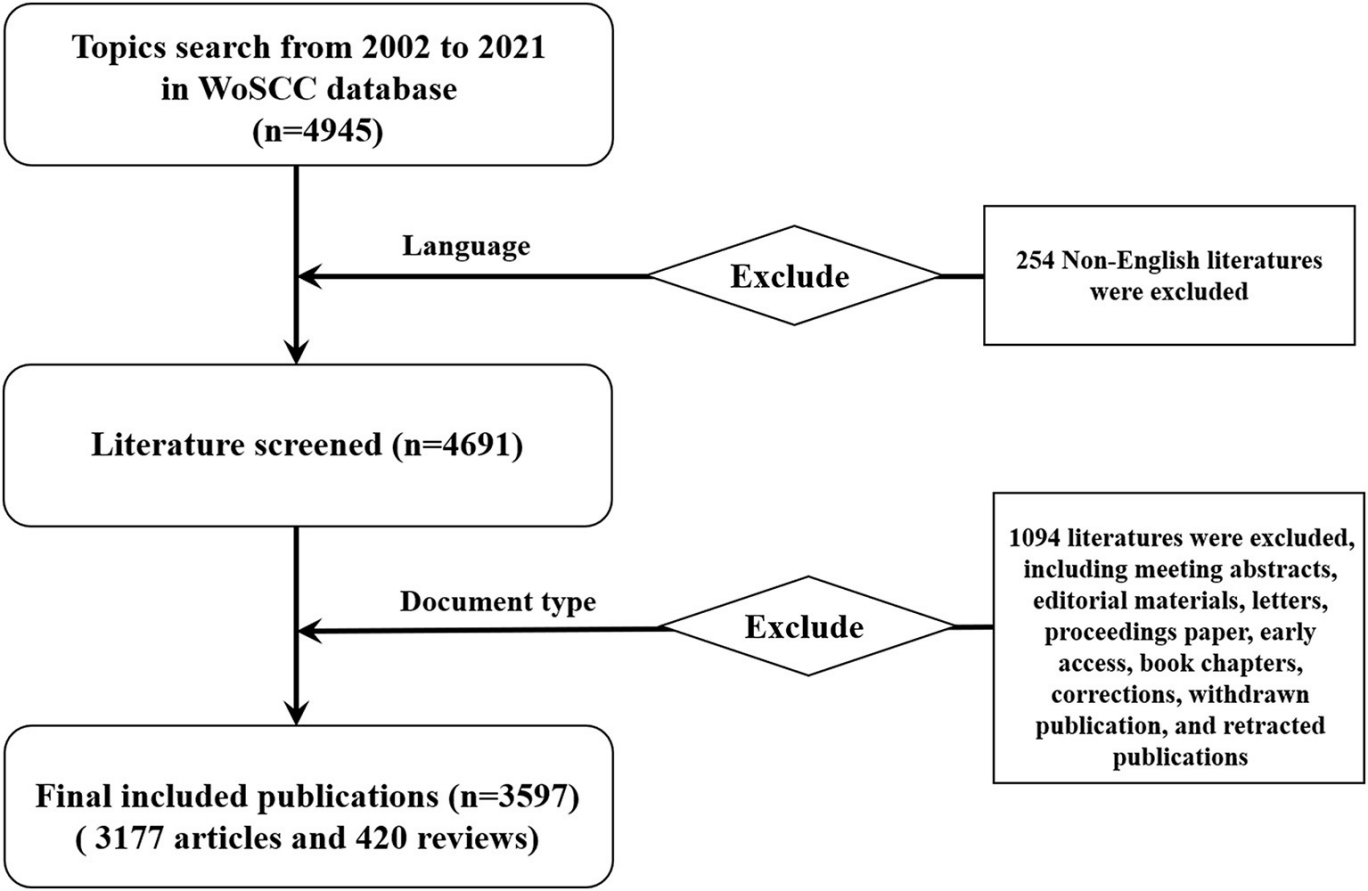
The flowchart of data screening.

We conducted the literature search on the Web of Science Core Collection (WoSCC) on pharmaceutical care in the past two decades (from 2002 to 2021). The search formula was as follows: TS = (“pharmaceutical care” OR “pharmaceutical service^*^,” and OR “pharmacy service^*^”). The publication language in this study was set to English. Of various document types, only articles and reviews with no duplication were considered. To avoid deviations from database renewal, we performed the literature retrieval on a single day (May 22, 2022). All information was collected in the format of text. And the number of publications and citations, titles, author information, institutions, countries/regions, keywords, journal, funding agencies, and references were collected for further bibliometric analysis. In total, 3,597 eligible publications were ultimately analyzed in the present study. The flowchart of data screening is shown in Figure 1.

### Statistical analysis

In the present study, VOSviewer (version 1.6.17) and CiteSpace (Version 5.8.R3) were employed to perform the bibliometric analysis. And Microsoft Excel (Office 2019) was applied to manage data and draw figures after data deduplication with CiteSpace. VOSviewer was used to conduct the co-authorship networks of cooperation among countries/regions, institutions, and authors, visualize the co-occurrence networks of keywords and the complex co-citation networks of references for revealing research trends. The size of nodes represents the number of publications, the thickness of lines symbols the strength of the link, and the color of nodes stands for different clusters or times. CiteSpace was applied to perform the burst analysis of keywords to detect new research trends in the research field of pharmaceutical care.

In addition, we employed H-index and the impact factor (IF) to quantify the academic impact of individuals and journals, respectively. H-index is a vital indication to evaluate the academic contribution of researchers and could predict their future scientific achievements (21, 22). IF is a leading indication for measuring the quality and impact of scientific journals (23). In this study, H-index of each author was obtained from WoSCC, and IF was acquired from 2020 Journal Citation Reports (JCR).

## Results

### An overview of publications in research of pharmaceutical care

**Figure 2:**
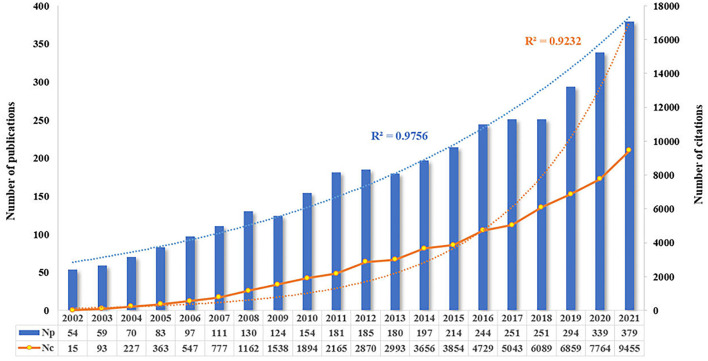
Annual output of publications and citations in the field of pharmaceutical care during the past two decades.

From the search strategy, the total number of publications (Np) concerning the research theme of pharmaceutical care published between 2002 and 2021 were 3,597 (3,177 articles and 420 reviews). The total number of citations (Nc) was 62,093, with a mean Nc per paper of 17.26. Figure 2 displays the annual Np and Nc related to pharmaceutical care research. The annual Np and Nc were significantly associated with years, and their correlation coefficient R^2^ reached 0.9756 and 0.9232, respectively. The annual Np increased almost seven times in the past two decades, from 54 papers in 2002 to 379 articles in 2021, indicating increased attention to pharmaceutical care. Meanwhile, the sensational growth rate of Nc per year during the survey also confirms the focus of strong interest in pharmaceutical care.

### Contributions of countries/regions to global publications

**Figure 3:**
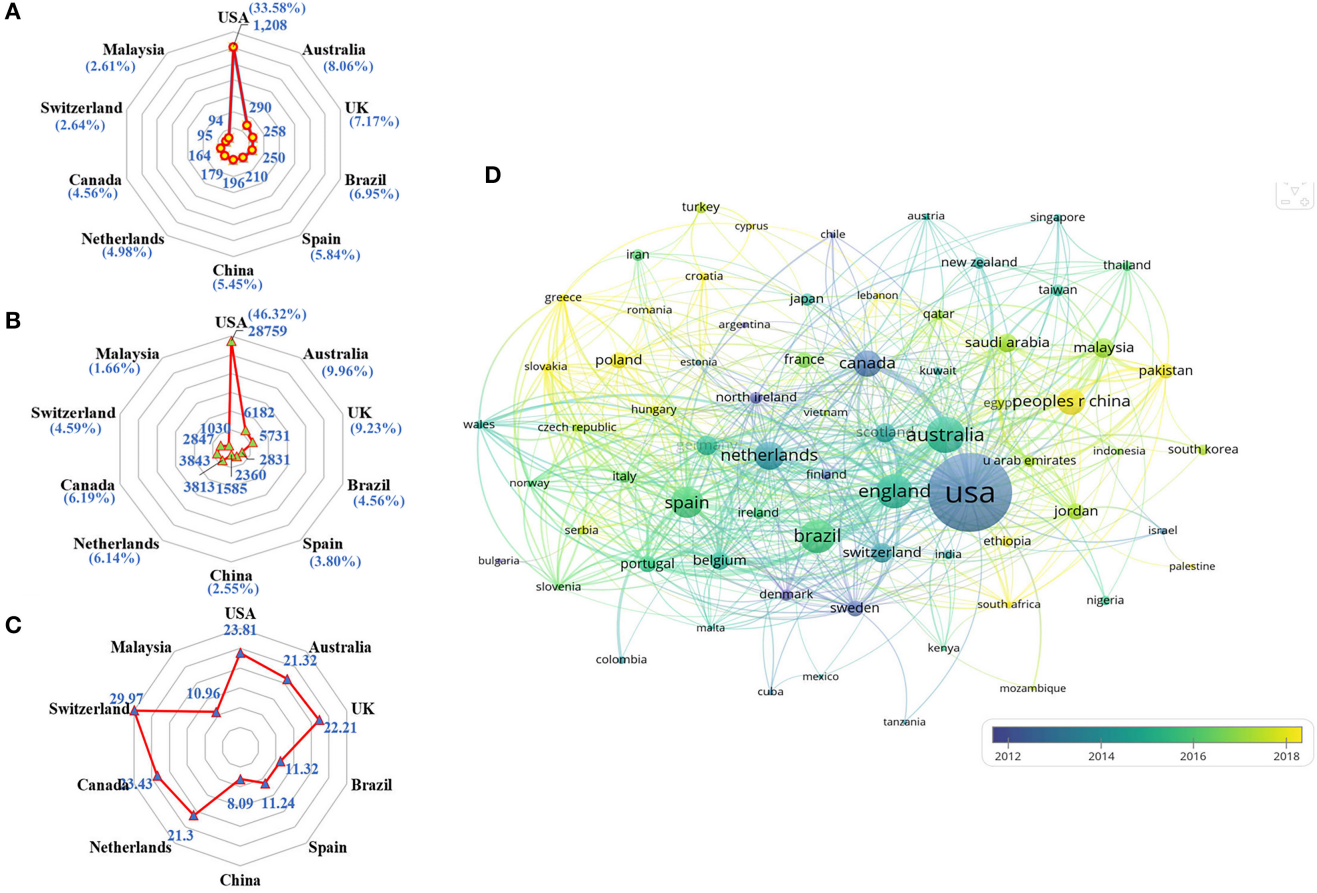
Co-authorship analysis of countries/regions. **(A)** Radar map of Np in the top 10 countries. **(B)** Radar map of Nc in the top 10 countries. **(C)** Radar map of average citations per paper in the top 10 countries. **(D)** Overlay map of countries/regions with more than 5 publications.

A total of 114 countries/regions published articles in this field. The top 10 productive countries generated 2,944 articles, accounting for 81.85% of the papers worldwide. The United States was the most productive country with the highest Np (*n* = 1,208, 33.58%), followed by Australia (*n* = 290, 8.06%) and the United Kingdom (*n* = 258, 7.17%). Besides, publications from the United States also owned the greatest Nc (*n* = 28,759, 46.32%), followed by those from Australia (*n* = 6,182, 9.96%) and the United Kingdom (*n* = 5,731, 9.23%). Of the top 10 productive countries/regions, 4 are in Europe, 2 in Asia, 2 in North America, 1 in South America, and 1 in Oceania. As shown in Figures 3A–C, the United States occupied the leading role in the field of pharmaceutical care from the perspectives of Np and Nc. At the same time, Switzerland possessed the highest number of average citations, indicating several viewpoints raised researchers' interest. In addition, 69 countries/regions with a minimum of five documents in co-authorship were analyzed with VOSviewer. The overlay visualization in Figure 3D shows widespread cooperation among observed countries/regions, and China and Malaysia have emerged in research of pharmaceutical care in recent 5 years.

### Analysis of institutions

**Figure 4:**
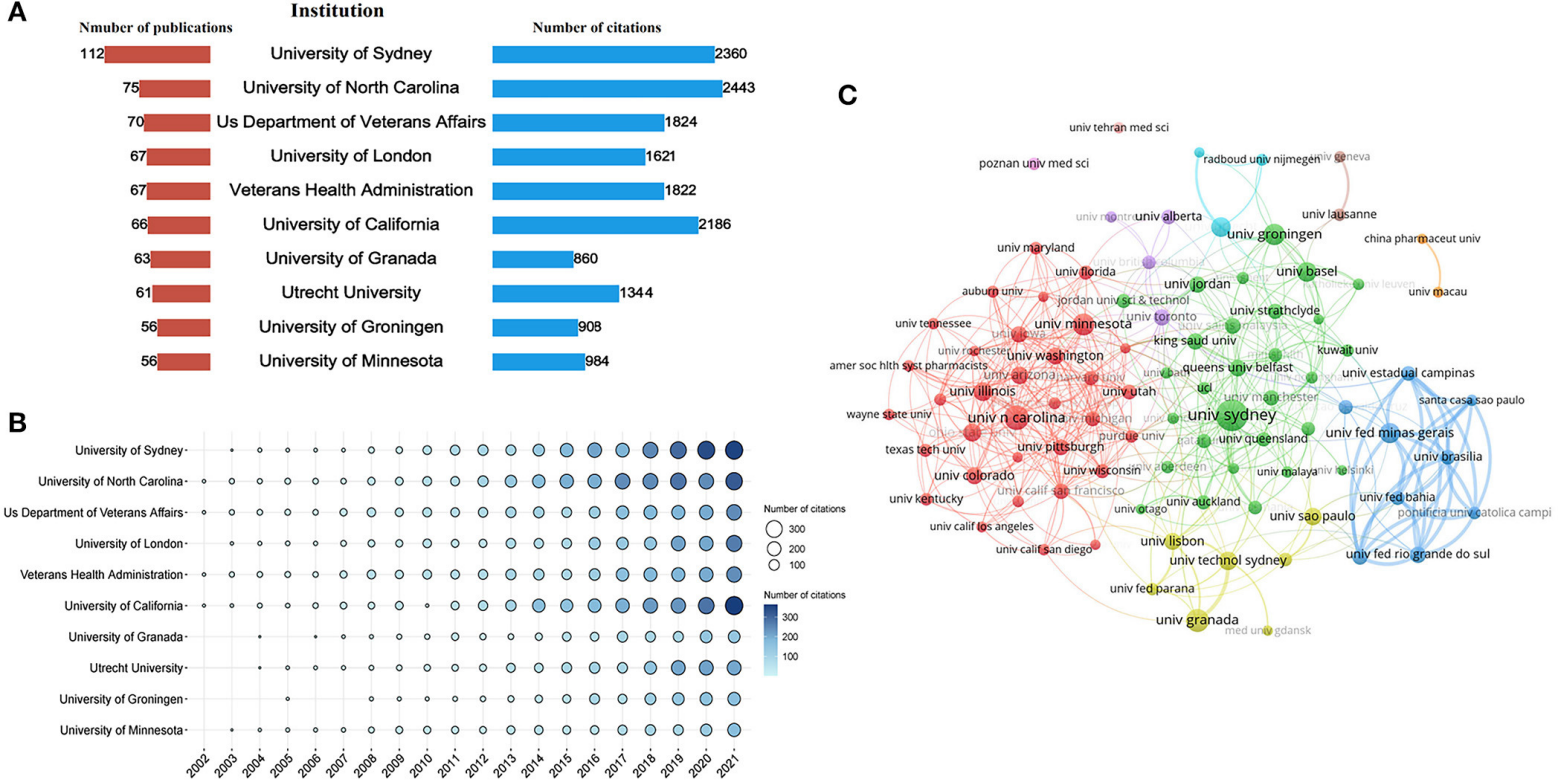
Co-authorship analysis of institutions. **(A)** Total Np and Nc of the top 10 institutions. **(B)** The yearly Nc of the top 10 institutions. **(C)** Cluster analysis of cooperation among institutes with more than 15 publications.

A total of 4,227 institutions were involved in this field. The top 10 institutions with the highest Np and Nc in research on pharmaceutical care are displayed in Figure 4A. The University of Sydney in Australia (*n* = 112) was the leading institution in terms of Np, followed by the University of North Carolina in the United States (*n* = 75), and the US Department of Veterans Affairs (*n* = 70). The Nc of the University of North Carolina (cited 2,443 times) was the highest, followed by the University of Sydney (cited 2,360 times), and the University of California (cited 2,186 times). The bubble chart in Figure 4B further illustrates the yearly Nc of the top 10 institutions. In addition, Figure 4C exhibits the cooperative relationship among 91 institutions that published a minimum of fifteen documents. It divides institutions into eight clusters. In the top 3 big cooperation groups, the University of Sydney, the University of North Carolina, and Universidade Federal de Minas Gerais played the central role that owned the closest cooperation with other institutions.

### Analysis of authors

**Figure 5:**
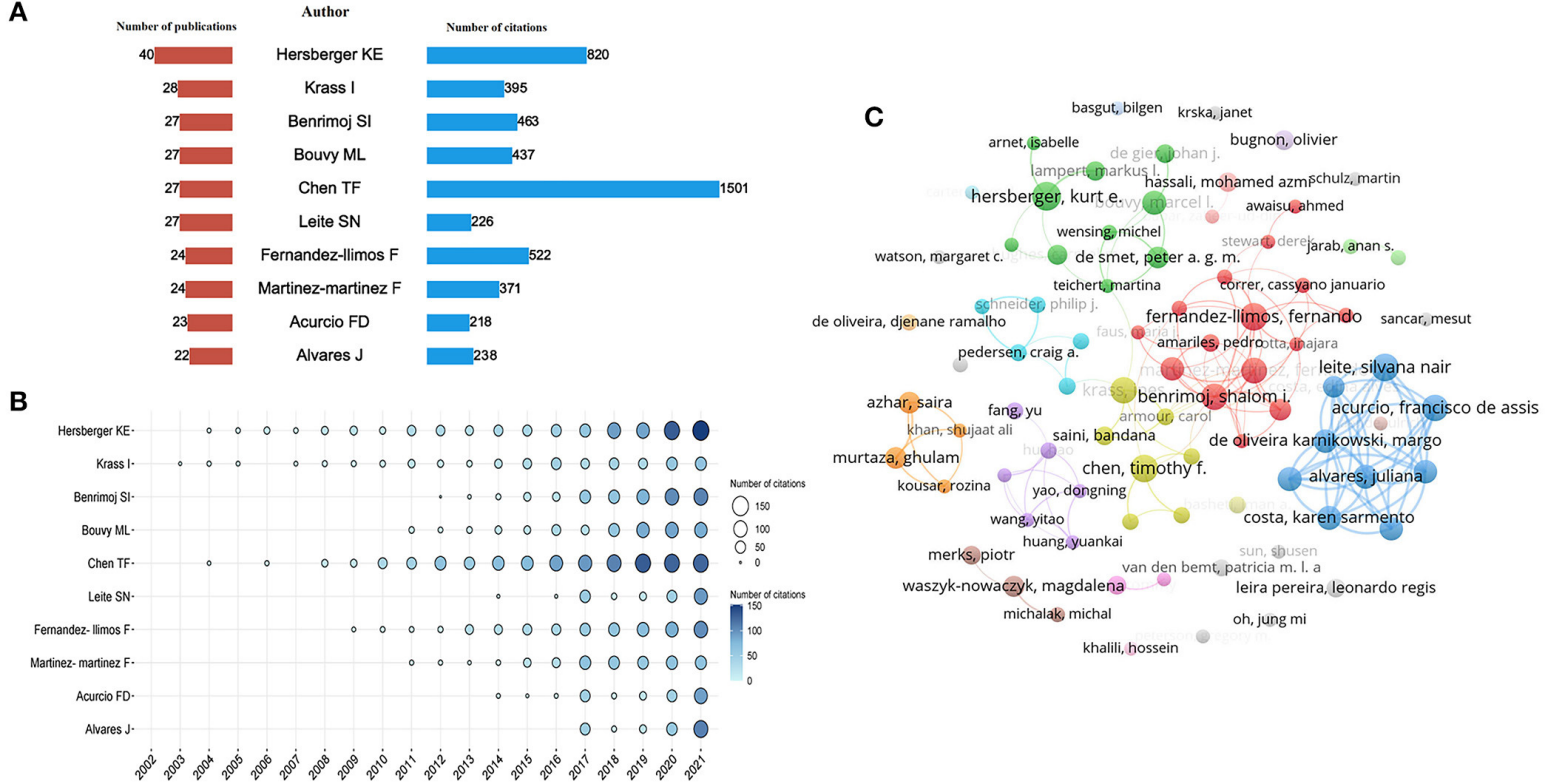
Co-authorship analysis of authors. **(A)** Total Np and Nc of the top 10 authors. **(B)** The yearly Nc of the top 10 authors. **(C)** Cluster analysis of cooperation among authors with more than 8 publications.

A total of 14,184 authors contributed to this field's publications from 2002 to 2021. The top 10 productive authors are listed in Table 1. They donated 269 papers which account for 7.48% of the total publications. Chen TF from the University of Sydney owned the highest H-index of 19. As shown in Figure 5A, the author Hersberger KE from the University of Basel published the most papers (*n* = 40), followed by Krass I from the University of Sydney (*n* = 28), Benrimoj SI from University of Technology Sydney (*n* = 27), Bouvy ML from Utrecht University (*n* = 27), and Chen TF (*n* = 27). At the same time, Chen TF also was the most cited author (cited 1,501 times), followed by Hersberger KE (cited 820 times), and Fernandez-llioms F from the University of Lisboa (cited 522 times). Figure 5B intuitively displays the yearly Nc of the top 10 authors. It demonstrated that Hersberger KE, Krass I, and Chen TF were early researchers engaged in pharmaceutical care for nearly 20 years with a significant impact. By tracking the specific research area of these authors and scanning their articles, we could quickly gain insight into the pharmaceutical care field. Hersberger KE and his colleagues mainly concentrate on medication safety, and concerned the pharmacogenetics in pharmaceutical care, which has been applied to assess the risk of medication use to better guide individualized drug administration and safe drug use in recent years (24–27). Krass I and Chen TF were more focused on the importance of pharmacists' roles in pharmaceutical care and recognized it has core effects (28–31).

**Table 1:** The top 10 productive authors in the field of pharmaceutical care.

**Rank**	**Author**	**Institution**	**Np**	**% of (3,597)**	**H-index**
1	Hersberger KE	University of Basel	40	1.11	15
2	Krass I	University of Sydney	28	0.78	12
3	Benrimoj SI	University of Technology Sydney	27	0.75	12
4	Bouvy ML	Utrecht University	27	0.75	13
5	Chen TF	University of Sydney	27	0.75	19
6	Leite SN	Universidade Federal de Santa Catarina	27	0.75	10
7	Fernandez-llimos F	University of Lisboa	24	0.67	13
8	Martinez-martinez F	University of Granada	24	0.67	10
9	Acurcio FD	Universidade Federal de Minas Gerais	23	0.64	10
10	Alvares J	Universidade Federal de Minas Gerais	22	0.61	11

In addition, Figure 5C visualized the co-authorship network of 82 authors who published more than eight documents. However, only 37 authors linked with the line in the network, which indicates the insufficient cooperation of authors in the research field of pharmaceutical care.

### Analysis of journals, funding agencies, and subject categories

**Figure 6:**
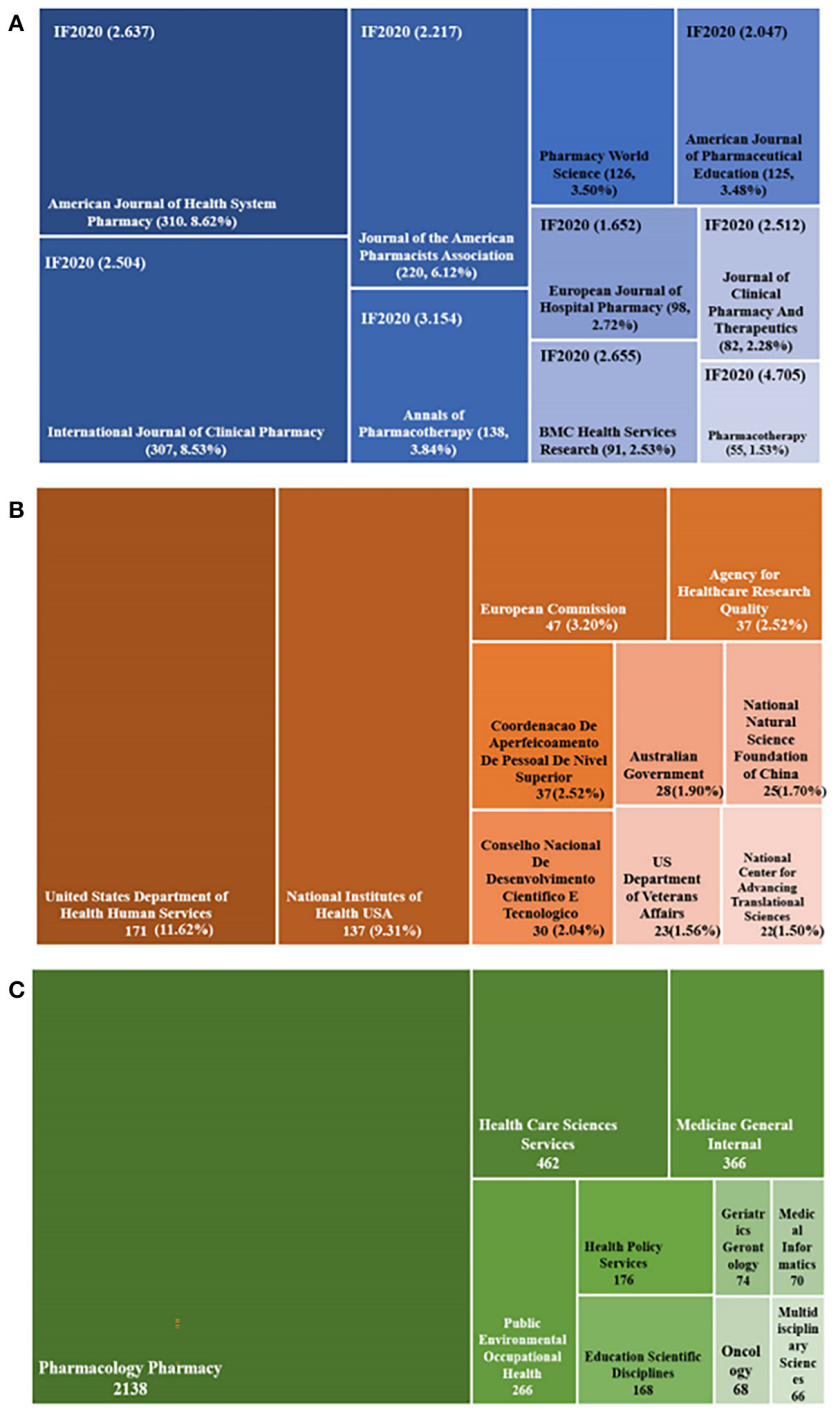
Distribution of journals, funding agencies, and subject categories. **(A)** The tree-map of top 10 journals. **(B)** The tree-map of funding agencies. **(C)** The tree-map of subject categories.

All papers were published in 610 academic journals. The top 10 most productive journals in the research field of pharmaceutical care are displayed in Figure 6A. Half of them were from the United States, three were from the United Kingdom, and two were from the Netherlands. About 43.15% of the articles were published in these journals. American Journal of Health System Pharmacy (*n* = 310, 8.62%) published the most articles, followed by the International Journal of Clinical Pharmacy (*n* = 307, 8.53%), and the Journal of the American Pharmacists Association (*n* = 220, 6.12%). Among those journals, Pharmacotherapy had the highest IF (IF_2020_ = 4.705) and ranked tenth in publication numbers (*n* = 55). The analysis of journals reflected areas that are interested in pharmaceutical care. Consistent with the core area of pharmaceutical care, pharmaceutical care obtained more attention from journals specializing in clinical pharmacy. Furthermore, pharmaceutical care continually receives attention from the pharmacotherapy field.

Only 1,471 of 3,597 publications owned support from the foundation. Half of the funding agencies came from the United States and sponsored 390 papers, occupying 26.51%, consistent with its high Np, Nc, and cooperation levels. More details are exhibited in Figure 6B. The tree-map in Figure 6C displayed the top 10 funding categories and the corresponding number of publications. The top three categories occupied over 80% of all the papers, which involved Pharmacology Pharmacy with 2,138 publications (59.44%), Health Care Sciences Services with 462 records (12.84%), and Medicine General Internal with 366 documents (10.18%).

### Analysis of co-cited references

**Figure 7:**
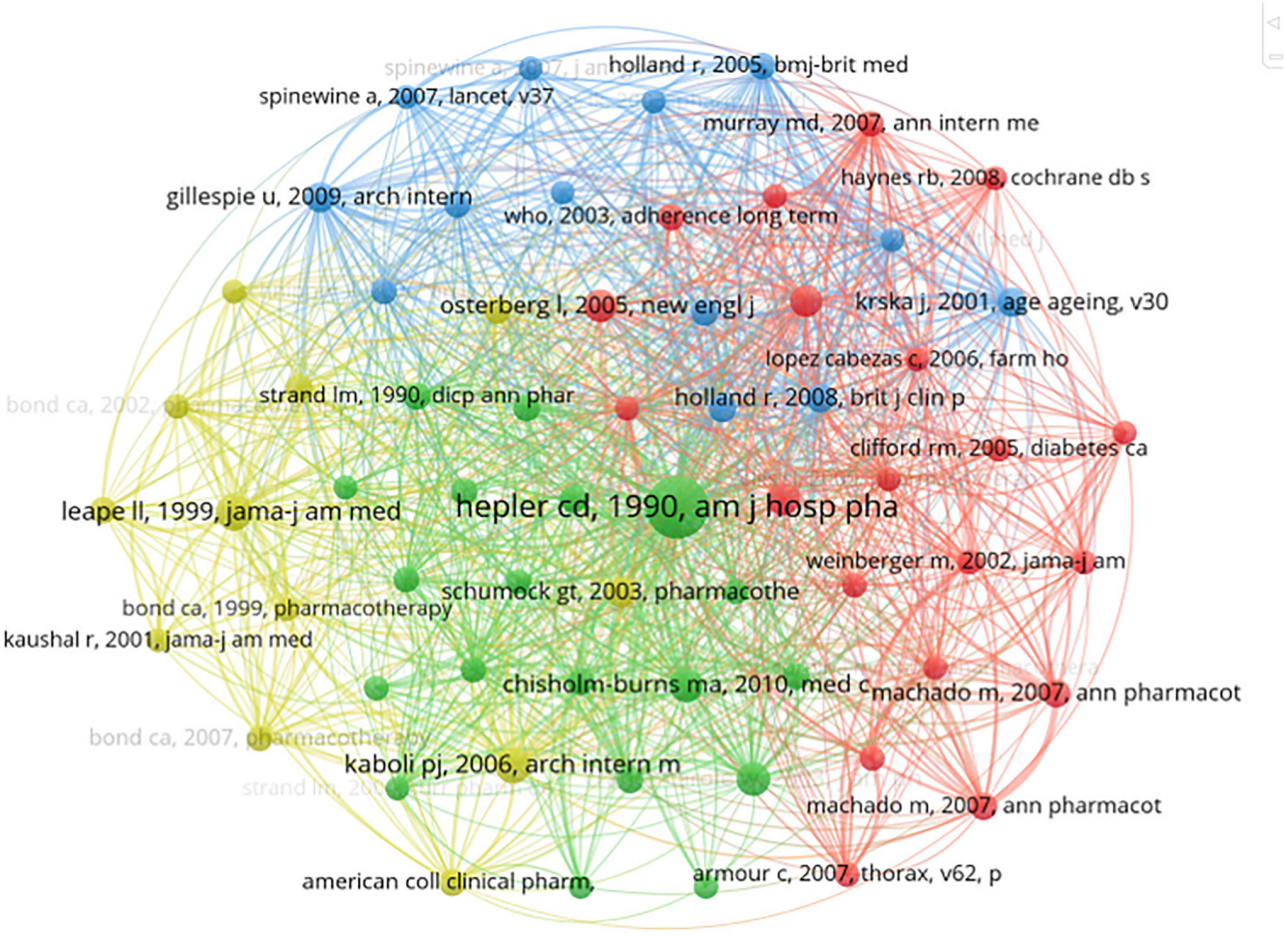
Co-occurrence analysis of co-cited references cited more than 40 times.

The co-citation analysis focuses on closely related research themes to a specific field. The retrieved papers cited a total of 62,093 references. The top 10 references with high co-citations are listed in Table 2. Each of them was co-cited over 70 times. Benefits driven by pharmaceutical care provided by pharmacists were their common topic, involving a lower rate of adverse events caused by prescribing errors, improved outcomes of drug therapy, increased medication adherence, and positive financial benefits of the clinical pharmacy service. Figure 7 shows the co-occurrence network of co-cited references cited more than 40 times. And 64 co-cited references were divided into four clusters symbolized with different colors. Cluster 1 (in red) included 20 references, which mainly evaluated the outcomes of pharmaceutical care using a randomized controlled trial. Cluster 2 (in green) contained 19 publications and primarily focused evolving of definitions and concepts in pharmaceutical care. Cluster 3 (in blue) consisted of 13 papers on pharmaceutical care for older people. Cluster 4 (in yellow) with 12 records looked at the role of clinical pharmacy in reducing adverse drug events and mortality rates.

**Table 2:** The top 10 co-cited references in the field of pharmaceutical care.

**Nc**	**Title**	**Author**	**Year**	**Journal**
439	Opportunities and responsibilities in pharmaceutical care	Hepler CD	1990	American Journal of Hospital Pharmacy
117	Pharmacist participation on physician rounds and adverse drug events in the intensive care unit	Leape LL	1999	JAMA
109	Clinical pharmacists and inpatient medical care: a systematic review	Kaboli PJ	2006	Archives of Internal Medicine
99	US Pharmacists' Effect as Team Members on Patient Care: systematic Review and Meta-analyses	Chisholm-Burns, MA	2010	Medical Care
97	The Asheville project: short-term outcomes of a community pharmacy diabetes care program	Cranor CW	2003	Journal of the American Pharmaceutical Association
93	Concurrent and predictive validity of a self-reported measure of medication adherence	Morisky DE	1986	Medical care
88	Adherence to medication	Osterberg L	2005	The New England Journal of Medicine
85	Effect of a pharmacy care program on medication adherence and persistence, blood pressure, and low-density lipoprotein cholesterol: a randomized controlled trial	Lee JK	2006	Jama
75	A comprehensive pharmacist intervention to reduce morbidity in patients 80 years or older: a randomized controlled trial	Gillespie U	2009	Archives of Internal Medicine
72	Evidence of the economic benefit of clinical pharmacy services: 1996–2000	Schumock GT	2003	Pharmacotherapy

### Analysis of keywords

**Figure 8:**
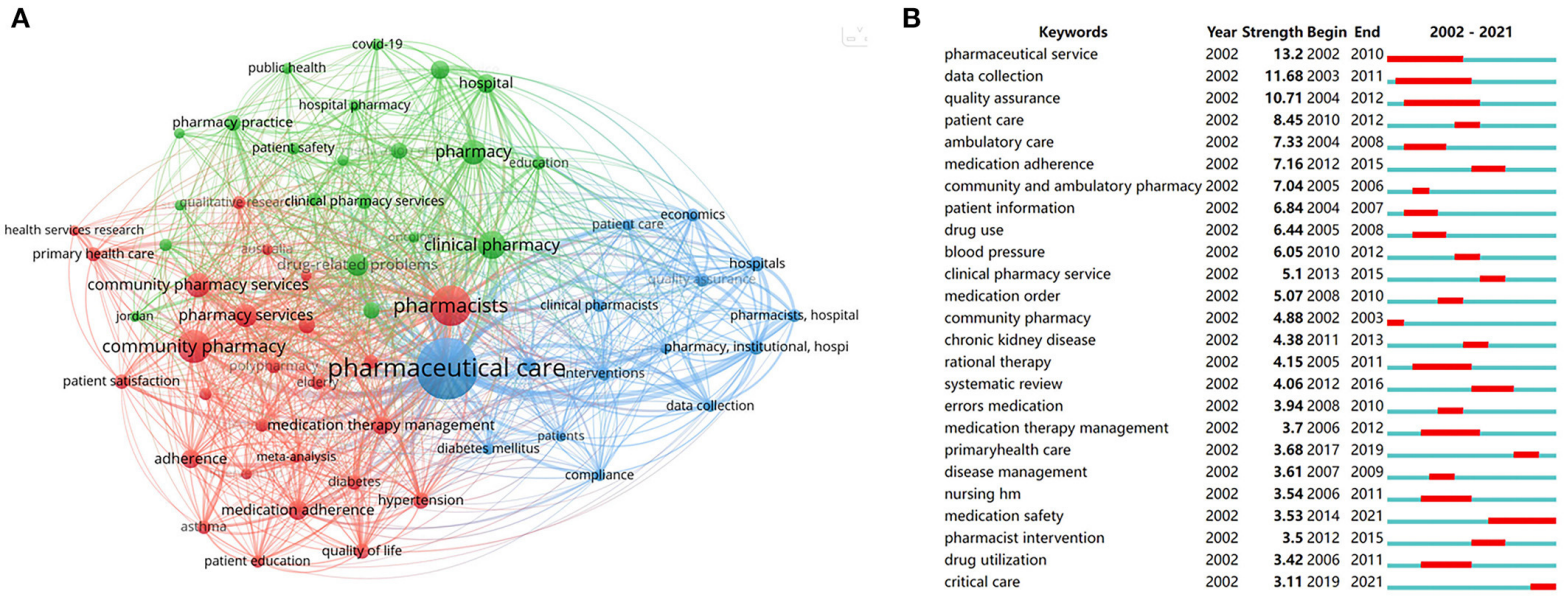
Analysis of keywords. **(A)** Co-occurrence analysis of keywords with the threshold over 30. **(B)** Burst analysis of the top 25 keywords.

A total of 5,187 authors' keywords were involved in the present study. Figure 8A identified 62 keywords with a threshold over 30 in the co-occurrence network. The top 10 keywords were as follows: pharmaceutical care (*n* = 1,006), pharmacists (*n* = 438), community pharmacy (*n* = 292), clinical pharmacy (*n* = 209), pharmacy (*n* = 166), community pharmacy services (*n* = 160), pharmacy services (*n* = 144), drug-related problems (*n* = 132), medication adherence (*n* = 104), and hospital (*n* = 97). Three kinds of colors represent different clusters of keywords. Pharmaceutical care in blue, including data collection, hospitals, and economics. Pharmacists in red, including community pharmacy, medication therapy management, and medication adherence. Clinical pharmacy in green, including patient safety, drug-related problems, and medication reconciliation. In addition, we found COVID-19 appeared in the green cluster.

The burst analysis of keywords in a 2-year slice from 2002 to 2021 was performed to reveal the evolution trend in pharmaceutical care. The blue lines stand for the time span. The red lines represent the burst period. As shown in Figure 8B, the strength of the top 25 keywords with the strongest bursts varied from 13.2 to 3.11. The term pharmaceutical service owned the highest burst strength from 2002 to 2010. The keywords medication safety burst from 2014 to 2021, illustrating an important topic and research hotspots in pharmaceutical care. More recently, the theme of critical care suddenly appeared in 2019, suggesting an emerging role of pharmaceutical care in urgent care.

Overall, keywords analysis could obtain the developmental trajectory of pharmaceutical care, including the main research areas, current research concerns, and future research trends. Our study reveals that medication safety is a core area of concern, and the target groups of pharmacy services gradually transfer to critical patients, indicating that critical care may be a hot area in future research.

## Discussion

We carried out a bibliometric analysis to investigate global trends and research frontiers in pharmaceutical care over the past 20 years in the present study. It found a strong interest in the pharmaceutical care field from researchers with increased annual publications. The United States occupied the leading role in pharmaceutical care in publications, citations and cooperation, which may not be separated from its strong support from funding agencies. Hersberger KE and Chen TF were the two most outstanding researchers in this field. However, academic cooperation among authors from different institutions was insufficient. Meanwhile, the IF values of journals published articles concerning pharmaceutical care is no more than five. It may mean a challenge for publishing high-impact articles in this field. We appeal for more cooperation from scholars and attention from influential journals in pharmaceutical care. In addition, analysis of co-cited references and keywords suggested that clinical pharmacy is an essential theme in pharmaceutical care. Terms of medication safety and critical care recognized by burst analysis of keywords also hint at the recent attention on clinical pharmacy.

1) Clinical pharmacy is an essential theme in pharmaceutical care

Pharmaceutical care is a concept started based on mercantile operations, and transformed into a clinical profession in the community and hospital (32). The clinical pharmacy was introduced during this period to change pharmaceutical care from product-oriented to patient-centered. At present, clinical pharmacy is defined as “a health science discipline where pharmacists provide patient care that optimizes medication therapy and promotes health, wellness, and disease prevention” (33). In general, clinical pharmacy includes all the services pharmacists perform in hospitals, community pharmacies, nursing homes, home-based care services, clinics and other settings where medicines are prescribed and used (34). Its primary contents could be classified into two broad aspects. One is supporting the implementation of national drug policies, including assessing medicine use, reviewing formularies, and managing the overall clinical risk associated with medication. The other is patient-specific services, including obtaining patients' medication histories, evaluating therapeutics, involving in medicine selection, ward rounds, provision of medicines information, and adverse drug reactions management (35).

Therefore, clinical pharmacy is an essential theme in pharmaceutical care. It has shown a potential to contribute significantly to pharmaceutical care and is responsible for ensuring that patients receive the right medicine at the right time through an efficient and economical system (36).

2) Medication safety is the primary goal of clinical pharmacy

Medication safety is an essential component of patient safety (37). As health systems expand clinical pharmacy services, assessment of drug risks and mitigation of medication errors have high priority in clinical pharmacy. Risks to medication safety risks are usually due to multiple factors, such as drug-related adverse reactions, illegal supply of prescription-only medication, self-medication, and so on (38). The inclusion of clinical pharmacists created multiple strategies to promote the safety of medicines, including collaborating in the development of a research protocol, reviewing as a member of an advisory committee, developing mechanisms that contribute to safety, and assuring compliance with local and national regulations and standards (39).

Currently, clinical pharmacists are engaged in providing drug information, drug therapy evaluation, drug therapy intervention, medication review, and medication reconciliation (40, 41). Multiple ways have been applied in their work to target potential risk factors and develop systems that ensure the safe use of medication. Specifically, the Human Factors Framework (HFF) (42), the Patient Safety and Clinical Pharmacy Services Collaborative (PSPC) (43) and the High Reliability Organizations (HROs) (44) were tools to help to reduce medication error rates. In addition, observational studies are essential to inform the safe use of medications and are generally divided into two types. One of the clinical databases contains electronic medical records entered for clinical use and patient monitoring, for example, the Clinical Data Analysis and Reporting System database (CDARS, Hong Kong) and the Clinical Practice Research Datalink (CPRD, United Kingdom). The second is administrative databases, including the Medicaid (United States) and the National Health Insurance Research Database (NHIRD, Taiwan). Besides, the self-controlled case series study (SCCS) and case-crossover study (CCO) is the fundamental and traditional methods that are also widely applied for observation and evaluation of drug safety and effectiveness (45).

Studies show that 30–60% of adverse drug reactions (ADR) can be prevented (46). Clinical pharmacists can provide appropriate medication counseling before treatment is initiated and during subsequent treatments. They can contribute by working with doctors, nurses and patients to improve the quality of ADR reporting and management. These could help in the early detection and prevention of ADR. Furthermore, based on the spontaneous reporting and active surveillance systems (47), the pharmacovigilance and drug and therapeutics committee could assist in monitoring the quality and safe use of medicines to improve medication safety (48). Another important part of the clinical pharmacist's role is medication reconciliation and supporting patient adherence (49), especially in geriatrics. Medication reconciliation at admission to the hospital reduces the prevalence of medication errors. The integrated cloud technology named PharmaCloud (Taiwan) is applied at a national level to optimize medication use during transitions of care (50).

Therefore, it can be seen that clinical pharmacy and clinical pharmacists play an important role in medicines safety, which can improve patient outcomes, reduce adverse drug events, and facilitate the safe and effective use of medication in patients (51).

Furthermore, besides clinical pharmacists, community pharmacists are the essential entities providing drugs to individual patients. The combination of location and accessibility offers people the convenience of consulting drugs and health professional advice from community pharmacists (52). It means community pharmacists may have more challenges in work compared with other health care professionals. Among them, ensuring drug safety was the top priority. Studies explored the medication safety problems in community pharmacies using the HFF. Medication safety problems were mainly categorized into the following aspects: commercial pressure on community pharmacies, illegal supply of prescription medication, lack of enforcement of regulations, communication failure, and self-medication (53, 54). Moreover, the suggestions for improving medication safety in community pharmacies included continuous education for community pharmacists and competency assessment focusing on medication safety, encouraging medication error and adverse drug reactions reporting, and promoting national patient safety initiatives (55).

3) Critical care and clinical pharmacy

Critical care is a high-risk medication area where vulnerable, acutely unwell patients are treated with intense therapies in a complex environment (56), which emerged in the 1930s and evolved into an intensive care unit (ICU) in the 1950s. Due to the acuity of their illness, multi-organ failure, medication exposure, and frequency of medication changes, these patients are vulnerable to medicines-related harm. Thus, critical care patients are at approximately twice the risk of medication errors compared to ward-based patients (57). Among them, approximately 60% of all medication errors occur during times of transition, and 66% of medication reconciliation errors result from failure to reconcile medications during transitions from 1 level of care to another (58).

To our knowledge, clinical pharmacists are positioned to be leaders in reducing medication errors, and assuming significant roles in critical care. They consulted/collaborated closely with the multidisciplinary team to undertake individual patient medication reviews, attend ward rounds, and provide professional support services (59). The involvement of clinical pharmacists has been shown to benefit medication safety, improve patient outcomes and reduce medicines expenditure (60). The Joint Task Force of the Society of Critical Care Medicine and the American College of Clinical Pharmacy also recommends that each ICU should employ a dedicated pharmacist (61). Later known as the critical care pharmacist (CCP), The United Kingdom also recommends 0.05–0.1 SCCP per level three patient (62). As early as 2000, the Society of Critical Care Medicine (SCCM) and the American College of Clinical Pharmacy (ACCP) published a position paper that defined the scope of critical care pharmacy services (63). Recently, that position paper was updated in 2020. This time delineates the activities of a critical care pharmacist and the scope of pharmacy services within the ICU (64). Clinical pharmacy has always continued to advance in critical care and is equally vital in the field of pandemics.

Pandemics and the large-scale outbreak of infectious disease can significantly impact morbidity and mortality worldwide with the potential to stress critical care resources (65). Such as Coronavirus Disease 2019 (COVID-19), had affected over 120,000 individuals in more than 80 countries, and resulted in more than 5,000 deaths worldwide (66). The impact on ICU resource was significant and required profession and precision of clinical pharmacy service under the rapidly changing clinical contexts. The CCP was involved in COVID-19 treatments and ensured that potential treatments were analyzed taking into account pharmacodynamics and pharmacokinetics and exploring optimal dosing regimens (67).

### Limitation

The present study provided a comprehensive overview of the global trends and research frontiers in pharmaceutical care over the past 20 years. Although bibliometric analysis is relatively more objective than traditional reviews, there are also several inevitable limitations. Firstly, we only included articles and reviews in English writing from the WOSCC database. Secondly, some new research papers may be omitted for their smaller number of citations. Lastly, the actual contribution of different authors or institutions could not be distinguished by VOSviewer due to its deficiency in analyzing the full texts of the publications simultaneously.

## Conclusion

The present bibliometric study revealed the developmental trends and tracked frontiers in the pharmaceutical care field during the past two decades. According to the analysis, the United States played the leading role in this research from multiple aspects, including publication, citations, funding agencies, and collaboration worldwide. The University of Sydney was the most contributed institution with the greatest number of publications in pharmaceutical care research. Hersberger KE from the University of Basel, the most productive author, and Chen TF from the University of Sydney, the author who owed the highest H-index and most citations, were two outstanding researchers who significantly impacted this field. However, the degree of cooperation among authors was insufficient. American Journal of Health System Pharmacy produced the most publications, while Pharmacotherapy had the highest IF in this field. The cluster networks of co-cited references and keywords suggested that clinical pharmacy was an essential theme in pharmaceutical care. Terms of medication safety and critical care recognized by burst analysis of keywords also hint at the recent attention on clinical pharmacy. It suggested that clinical pharmacy will become a core discipline of public concern for pharmaceutical care in the future, and pharmacists will become the main responsible for safe drug use, whether in the hospital sector or community pharmacy. Thus, pharmacists should have comprehensive and extensive professional knowledge and skills for better pharmaceutical care. This study could provide a comprehensive overview and valuable reference for future researchers and practitioners in the research field of pharmaceutical care.

## Data availability statement

The original contributions presented in the study are included in the article/supplementary material, further inquiries can be directed to the corresponding authors.

## Author contributions

BZ conceived the study. YW and YR collected data and wrote the manuscript. YY and YL rechecked data. ZL revised the manuscript. All authors read and approved the final manuscript.

## Funding

This study was supported by the National Leading Talents Support Plan of Traditional Chinese Medicine Qihuang Scholar Plan (No. 1040063320004); the National Special Support Plan for High-level Talents (Plan of Ten Thousand People) Famous Teacher Program (No. 2020063320001).

## Conflict of interest

The authors declare that the research was conducted in the absence of any commercial or financial relationships that could be construed as a potential conflict of interest.

## Publisher's note

All claims expressed in this article are solely those of the authors and do not necessarily represent those of their affiliated organizations, or those of the publisher, the editors and the reviewers. Any product that may be evaluated in this article, or claim that may be made by its manufacturer, is not guaranteed or endorsed by the publisher.
